# Differences in Estimated vs Measured GFR Associations with Cognitive Function in African American Older Adults

**DOI:** 10.1093/geroni/igaf122.2902

**Published:** 2025-12-31

**Authors:** Harshin Sanjanwala, Xiaoqian Zhu, B Gwen Windham, Srishti Shrestha, Michael Griswold, Andrew Rule, Thomas Mosley, Kevin Sullivan

**Affiliations:** University of Mississippi Medical Center, Jackson, Mississippi, United States; University of Mississippi Medical Center - The MIND Center, Jackson, Mississippi, United States; University of Mississippi Medical Center - The MIND Center, Jackson, Mississippi, United States; University of Mississippi Medical Center - The MIND Center, Jackson, Mississippi, United States; University of Mississippi Medical Center - The MIND Center, Jackson, Mississippi, United States; Mayo Clinic, Rochester, Minnesota, United States; University of Mississippi Medical Center - The MIND Center, Jackson, Mississippi, United States; University of Mississippi Medical Center - The MIND Center, Jackson, Mississippi, United States

## Abstract

Impaired kidney function is a risk factor for cognitive decline and dementia, but most studies use serum creatinine-based equations to estimate kidney function as estimated glomerular filtration rate (eGFR); these equations include age, also a strong driver of cognitive impairment, and produce eGFR estimates with substantial error compared to gold-standard objective measures of GFR (mGFR). The purpose of this study was to determine eGFR and mGFR associations with cognitive function in African American adults, a population disproportionately impacted by both chronic kidney disease and dementia. We conducted a cross-temporal analysis of 368 participants (Age: 66.4 ± 7.4y, 75% Female) in the Genetic Epidemiology Network of Arteriopathy study with eGFR (CKD-EPI 2021 Creatinine Race-Free Equation) and mGFR assessed. eGFR was assessed using the CKD-EPI 2021 Creatinine Race-Free Equation and mGFR (urinary clearance of non-radiolabeled iothalamate). Memory (Rey Auditory Verbal Learning Test; RAVLT) and executive function (Digit Symbol Substitution Test; DSST) were assessed. Linear regression models were used to relate eGFR, mGFR, and serum creatinine to cognitive function with and without adjustment for age and sex. In unadjusted models, eGFR was positively associated with DSST scores (β = 0.19, 95%CI:0.11, 0.27) and RAVLT scores (β = 0.10, 95%CI:0.04, 0.16). mGFR was marginally associated with DSST scores (β = 0.07, 95%CI:-0.00, 0.14), but not RAVLT scores (β = 0.03, 95%CI:-0.01, 0.07). Associations of serum creatinine with cognitive function were mostly null to marginal. Age and sex adjustment greatly attenuated all associations. The observed differential renal function associations with cognition suggest age may largely explain prior reported associations.

